# The relationship between interpersonal emotional regulation and psychological resilience in nursing undergraduates: a cross-sectional study and network analysis

**DOI:** 10.3389/fpsyg.2025.1546042

**Published:** 2025-03-07

**Authors:** Panpan Huai, Panyu Liu, Xiaomeng Wang, Yunan Wang, Hui Yang, Meiqin Yan, Jinli Guo

**Affiliations:** ^1^School of Nursing, Shanxi Medical University, Taiyuan, China; ^2^School of Nursing, Peking University, Beijing, China; ^3^Children's Hospital Affiliated to Shanxi Medical University, Taiyuan, China; ^4^The Second Hospital of Shanxi Medical University, Taiyuan, China

**Keywords:** interpersonal emotion regulation, psychological resilience, nursing undergraduates, network analysis, cross-sectional study

## Abstract

**Background and purpose:**

Nursing students are more likely to have mental health problems due to the influence of many social environment factors, such as busy professional courses, difficult balance of learning and practice and complex and changeable clinical work environment. Psychological resilience, as a well-known protective factor, plays an important role in maintaining students’ mental health. At the same time, from the perspective of social interaction, the particularity of close interpersonal contact (including colleague relationship, nurse–patient relationship, etc.) in clinical practice makes interpersonal emotion regulation ability of nursing students have great significance. The emotion-regulation framework suggests a strong link between psychological resilience and emotional regulation. However, the correlation and function between the various components of psychological resilience and interpersonal emotional regulation are still unclear. Network analysis can realize the objective analysis and prediction of complex symptoms. This study investigated the network structure of interpersonal emotional regulation ability and psychological resilience in nursing undergraduates, aiming to identify central and bridge nodes and provide suggestions for precise intervention of interpersonal emotional regulation ability and psychological resilience in nursing undergraduates.

**Methods:**

A total of 1,948 nursing undergraduates were included in our study. Interpersonal Regulation Questionnaire (IRQ) was used to assess interpersonal emotional regulation. Psychological Resilience levels were assessed using The Connor-Davidson Resilience Scale (CD-RISC). Network analysis is used for statistical analysis.

**Results:**

Network analysis revealed that IRQ14 (Enjoy being with friends), IRQ11 (Like to share), and CD-RISC11 (Believe in achieving goals) are core nodes of the interpersonal Emotional regulation-psychological resilience network. IRQ14 (Enjoy being with friends), CD-RISC2 (intimate relationship), and CD-RISC3 (The help of fate) are bridging nodes linking interpersonal emotional regulation and psychological resilience. In addition, the network structure is slightly different between different grades (upper and lower grades).

**Conclusion:**

The relationship between core nodes and bridge nodes revealed in this study (including interpersonal relationship, positive emotion, optimistic personality and self-efficacy, etc.) may provide help for the accurate intervention of students’ interpersonal emotion regulation ability and psychological resilience level.

## 1 Introduction

Mental health problems such as anxiety and depressive symptoms are common among college students worldwide. According to the World Health Organization World Mental Health Surveys, one in five (20.3%) university students in 21 countries suffer from a mental disorder, which may be related to mental health problems during college (Auerbach et al., 2016). Due to the influence of many social and environmental factors, such as busy professional courses, heavy clinical training tasks, complicated and changeable clinical work environment and difficult balance of learning and practice, nursing students are faced with greater challenges after entering school (Luo et al., 2019; Yi et al., 2022). In addition, nursing students generally have a low sense of professional identity and professional honor. The low level of professional identity will lead to errors in students’ understanding of the significance of professional work and social impact, which is prone to problems such as insufficient preparation for clinical practice and high stress level (Al-Noumani et al., 2024). At the same time, nursing students have the particularity of female students in gender, and female students have lower psychological adjustment ability than male students (Ma, 2024). Studies have reported that the majority of nursing students have moderate to severe levels of stress, and nearly 25% of students show moderate to very severe negative emotional states (Rosenthal et al., 2021; Kupcewicz et al., 2020). Compared with other majors, nursing students are more likely to have mental health problems such as anxiety and depression (Tung et al., 2018; Tang et al., 2024), and long-term exposure to such environments can lead to a variety of negative outcomes, including lower academic performance, lower quality of life, and strained interpersonal relationships (Sonmez et al., 2023).

Psychological resilience is a well-known protective factor in maintaining mental health, which refers to an individual’s ability to adjust, adapt positively and cope successfully in the face of setbacks and stress (Troy et al., 2023). Previous studies have shown that psychological resilience can effectively reduce the negative impact of study stress on nursing students and play a moderating role between academic stress and quality of life (Berdida and Grande, 2023). Therefore, improving the level of psychological resilience is of great significance to maintain the mental health of nursing students. At present, researchers often use stress coping methods to explain the differences in people’s psychological resilience levels, but this method has limitations such as broad classification of coping strategies, insufficient assessment methods and neglect of individual emotional states (Troy et al., 2023). In recent years, it is worth noting that emotional regulation methods, to a certain extent, are products of coping methods, and their relationship with individual psychological resilience has attracted wide attention of scholars. Emotion regulation method refers to the behavior of individuals to manage and control their own emotional experience (McRae and Gross, 2020). Troy et al. (2023) integrated coping approach and emotion-regulation approach, and put forward the influence regulation framework, which theoretically pointed out the close relationship between the level of individual psychological resilience and emotional regulation strategies. In addition, according to the Broaden-and-build Theory, psychological resilience is the result of the accumulation of positive emotions, which plays an important function in the construction of mental resources (Gao and Tong, 2010). Thus, it is necessary to explore the relationship between students’ psychological resilience and emotional regulation. Emotion regulation includes two aspects: individual emotion regulation and interpersonal emotion regulation (Hu et al., 2020). Interpersonal emotion regulation pays more attention to the social interaction between individuals, which refers to the process in which individuals consciously influence their own emotions or those of others through social interaction (Zaki, 2020). From the perspective of social interaction, the phenomenon of interpersonal emotional regulation accords with People’s Daily life experience and intuition, and as we have described before, nursing students have to deal with close interpersonal contact (including colleague relationship, nurse–patient relationship, etc.) during clinical practice and internship. These reasons make interpersonal emotional regulation ability play a more important role in nursing students. Previous research has found that people are more susceptible to negative emotions when they lack social interaction or when they are ostracized and isolated (Wang et al., 2024; Donker et al., 2020), and difficulty regulating interpersonal emotions has been shown to be a risk factor for disorders such as social anxiety, obsessive-compulsive disorder, and depression (Haliczer et al., 2021; Howard and Cheavens, 2023). Therefore, in view of the potential connection between psychological resilience and emotional regulation and the importance of interpersonal emotional regulation for nursing students, it is necessary to study the relationship between interpersonal emotional regulation and psychological resilience of nursing students, so as to provide reference for future intervention studies.

At present, most researchers regard psychological resilience and emotional regulation as a whole and explore the effects of emotional regulation and mental resilience on each other at the overall level within individuals. Common models include intermediary models and predictive models, such as the studies of Vaughan et al. (2019), Gu and Xu (2022). The complete research on the interrelationship between the internal structure and component elements of emotion regulation and mental toughness is still lacking, and only one study was found to analyze the network structure of emotion, emotion regulation and mental toughness in Chinese males (Guo et al., 2023). In terms of interpersonal emotion regulation from the perspective of social interaction, researchers pay more attention to the relationship between interpersonal emotion regulation ability and mental health (positive emotion, negative emotion and mental illness, etc.), social ability (perceived support, interpersonal relationship, etc.) and individual behavior (Internet addiction, overeating) (Williams et al., 2018; Christensen, 2019; Christensen et al., 2020), etc. However, studies on the relationship between interpersonal emotion regulation and mental toughness are still lacking. Network analysis is a method to visually explore the complex relationship between different components of a mental construct or related behaviors, and it is widely used in psychology and psychiatry (Galderisi et al., 2018; Wei et al., 2021). According to the network theory, the psychological concept is regarded as a whole network, through the discussion of each node (each component) and edge (the relationship between components) in the network, and then determine the core characteristics of a psychological concept, including the centrality of nodes, bridge function, etc. (Epskamp et al., 2018; Borsboom, 2017). In this case, accurately describing these interactions and locating the most important nodes in the network, with central nodes as potential targets, can enable precise interventions. Therefore, from the perspective of network, the importance of each component (that is, each item) in psychological resilience and interpersonal emotional regulation is different. Instead of using a simple overall score to explain the relationship between psychological resilience and interpersonal emotional regulation, researchers should pay attention to the interconnections and effects of each component.

This study examined the statistical network structure of interpersonal emotional regulation ability and psychological resilience of nursing undergraduates, and identified the centers and bridge nodes aimed at improving interpersonal emotional regulation ability and psychological resilience of nursing undergraduates. In addition, we identified differences in the association between different grades (upper and lower grades).

## 2 Methods

### 2.1 Study design and data acquisition

A cross-sectional correlation study design was used in this study. Four undergraduate universities in Taiyuan, Shanxi Province were selected to recruit nursing undergraduates aged over 18 from January to March 2024. Inclusion criteria: nursing undergraduates; Volunteer to participate in this study; Exclusion criteria: Having a mental illness or taking psychoactive drugs; He is currently involved in other psychological intervention research. Sample size calculation: The sample size of this study is estimated according to the requirements of Pairwise Markov Random Field (PMRF) by common network analysis models (Niu et al., 2025). The parameters to be estimated by PMRF are as follows: Threshold Parameters: indicates the threshold or critical value of each node (calculation formula: n = N, N refers to the number of nodes in the network model); Pairwise Association Parameters: indicates the association strength or dependence between node pairs (calculation formula: n = N (n−1)/2, N refers to the number of nodes in the network model). Required sample size = threshold parameters + pairwise association parameters. There are two scales in this study: The Connor-Davidson Resilience Scale (CD-RISC-25) and Interpersonal Regulation Questionnaire (IRQ-16). The total number of items in the scale is 41, and the required sample size is 861 cases according to the formula, so the sample size of this study is at least 861 cases. In the end, a total of 1,948 nursing undergraduates were enrolled.

Data was collected anonymously using an online survey platform. Conducted using uniform instructions to allow researchers to introduce nursing undergraduates to the purpose, significance, and filling requirements of this study. After data collection, all items of the questionnaire were carefully reviewed and participants were asked to complete incomplete responses (if any) in a timely manner.

The Ethics Committee of Shanxi Medical University approved the study (2024SJL74). All study participants gave informed consent and participated voluntarily. The questionnaire was submitted anonymously and participant information is confidential.

### 2.2 Measurement of variables

#### 2.2.1 Interpersonal regulation questionnaire (IRQ)

IRQ is a 16-item self-report questionnaire, which is composed of four aspects: negative-tendency (5 items), negative-efficacy (3 items), positive-tendency (4 items), and positive-efficacy (4 items), and is used to assess an individual’s level of interpersonal emotion regulation (Williams et al., 2018). Participants were asked to recall specific events from the past. The seven-point Likert scale was adopted: completely disagree = 1, disagree = 2, somewhat disagree = 3, both agree and disagree = 4, somewhat agree = 5, agree = 6, fully agree = 7. A higher IRQ score indicates higher interpersonal emotional regulation, with an overall score ranging from 0 to 112. The Chinese version was used. This version proved to be useful for assessing the level of interpersonal emotional regulation ability in Chinese students (Liao et al., 2023). The questionnaire in this study had excellent internal consistency (Cronbach’s *α* =0.85).

#### 2.2.2 The Connor-Davidson resilience scale (CD-RISC)

CD-RISC is a 25-item self-report questionnaire consisting of three aspects: tenacity (13 items), optimism (4 items), and strength (8 items). Used to assess an individual’s level of mental flexibility (Connor and Davidson, 2003). Using a five-point Likert scale, participants were asked to rate how often each outcome item occurred: never = 1, rarely = 2, sometimes = 3, often = 4, and always = 5. A higher CD-RISC score indicates a higher level of psychological resilience, with an overall score ranging from 0 to 100. The Chinese version was used. The version proved to be useful for assessing the level of psychological resilience among Chinese students (Yu et al., 2011). The questionnaire in this study had excellent internal consistency (Cronbach’s α = 0.96).

### 2.3 Data analysis

We used R package qgraph (version 1.9.8) and Gaussian Graph Model (GGM) for network analysis of interpersonal emotional regulation and psychological resilience (Epskamp et al., 2018). The regularized random correlation network is built using the GGM model algorithm, which is based on the glasso (Graph Minimum Absolute Contraction and Selection Operator) process (Epskamp and Fried, 2018). Graph LASSO is a statistical regularization technique in which all edges are precisely shrunk to zero to reduce pseudo-correlations to produce a concise and sparse network that is easier to interpret and more stable (Friedman et al., 2008). On the other hand, the Extended Bayesian Information Criterion (EBIC) is used to select the best fit model. In a network, each object represents a node, and the connections between objects are called edges. In the graph, a blue edge indicates a positive partial correlation, while a red edge indicates a negative partial correlation. The thickness of the edges indicates the strength of the relationship between the nodes.

We calculate the expected impact of each node through R package qgraph to determine which nodes are more central or influential in the network. The expected impact (EI) is calculated as the sum of all the original values of the weights of the edges connected to the node. Compared with the traditional central index, calculating the expected impact is more suitable for network structures that contain both positive and negative associations. The higher the expected impact (EI) of a node, the higher the significance of the symptom in the network (Robinaugh et al., 2016). In addition, we calculated the bridge expected impact for each node with the R package network tools to uncover bridge symptoms as a pathway linking interpersonal emotional regulation and psychological resilience symptoms. Similar to expected influence, the Bridge Expect Influence (BEI) is calculated by summing a node’s edge weights, but only edges that connect nodes from one community with the other are counted. The larger the node bridge expected Impact (BEI), the more likely it is to activate the opposing community (Jones et al., 2021). The existing network has two node communities: one community for psychological resilience (25 projects from CD-RISC) and the other for interpersonal emotional regulation (16 projects from IRQ). We also assessed the predictability of each node with R package mgm. Nodes with high predictability are thought to be susceptible to nearby nodes. In order to control nodes with good predictability, it may be helpful to focus on relevant nodes.

R package bootnet (version 1.5) was used to evaluate the accuracy and resiliency of network estimates (Epskamp et al., 2018). First, we evaluate the accuracy of the edge weights by calculating the 95% confidence interval (CI) of the edge weights using non-parametric bootstrap. Secondly, the correlation stability (CS) coefficients were calculated using self-lifting sampling for a subset of participants to assess the stability of the node prediction effects and the node bridge prediction effects. It is recommended that the value of CS coefficient should not be lower than 0.25, and preferably higher than 0.50.

## 3 Results

### 3.1 Study sample

A total of 1,948 nursing undergraduates were included in the analysis. The mean age was 19.64 (SD = 1.53) years, including 1,176 students in the lower grades and 772 students in the upper grades. The average IRQ score of the whole sample was 19.21 (SD = 3.74), and the average CD-RISC score was 94.20 (SD = 16.40). Table 1 shows the abbreviations, averages, standard deviations, and predictability of all items on CD-RISC and IRQ.

**Table 1:** Descriptive statistics of the IRQ-16 and CD-RISC-25 items.

Items	Abbreviation	Mean	SD	Predictability
IRQ1	Seek company	4.32	1.45	0.640
IRQ2	Tell trouble	3.93	1.46	0.623
IRQ3	Need help	4.39	1.40	0.709
IRQ4	Express one’s emotion	4.34	1.44	0.656
IRQ5	Need attention	4.70	1.31	0.727
IRQ6	Appreciate support	5.51	1.23	0.509
IRQ7	Need to care	5.36	1.23	0.524
IRQ8	Appreciate help	5.55	1.20	0.467
IRQ9	Tell the going well things	4.61	1.35	0.541
IRQ10	Tell good things	4.67	1.33	0.475
IRQ11	Like to share	4.68	1.34	0.449
IRQ12	Celebrate the good things	5.15	1.22	0.623
IRQ13	Happier with friends	4.89	1.29	0.620
IRQ14	Enjoy being with friends	5.04	1.23	0.520
IRQ15	Stay around other people	4.66	1.38	0.683
IRQ16	Stay with the people you know	5.06	1.26	0.577
CD-RISC1	Adapt to the change	3.87	0.77	0.533
CD-RISC2	Intimate relationship	3.95	0.82	0.580
CD-RISC3	The help of fate	3.64	0.86	0.658
CD-RISC4	Handle things	3.64	0.80	0.578
CD-RISC5	Handle new challenges	3.80	0.80	0.491
CD-RISC6	See the humor	3.75	0.82	0.521
CD-RISC7	Become stronger	3.86	0.77	0.450
CD-RISC8	Self-regulation	3.75	0.80	0.536
CD-RISC9	Nothing comes of nothing	3.86	0.78	0.557
CD-RISC10	Do one’s best	3.93	0.78	0.442
CD-RISC11	Believe in achieving goals	3.88	0.77	0.413
CD-RISC12	Do not give up	3.84	0.80	0.479
CD-RISC13	Ways to get help	3.70	0.80	0.562
CD-RISC14	Concentrate	3.64	0.83	0.570
CD-RISC15	Take a lead role	3.59	0.87	0.629
CD-RISC16	Not easily defeated	3.85	0.78	0.428
CD-RISC17	A strong man	3.87	0.79	0.444
CD-RISC18	Make tough decisions	3.60	0.85	0.634
CD-RISC19	Deal with pain	3.80	0.79	0.527
CD-RISC20	Follow one’s instincts	3.56	0.81	0.758
CD-RISC21	Have a clear goal	3.78	0.81	0.499
CD-RISC22	Control one’s life	3.75	0.80	0.507
CD-RISC23	Love challenges	3.56	0.89	0.626
CD-RISC24	Strive to reach the goal	3.85	0.78	0.465
CD-RISC25	Take pride in achievement	3.88	0.82	0.554

### 3.2 Network structure and centrality measure analysis

**Figure 1:**
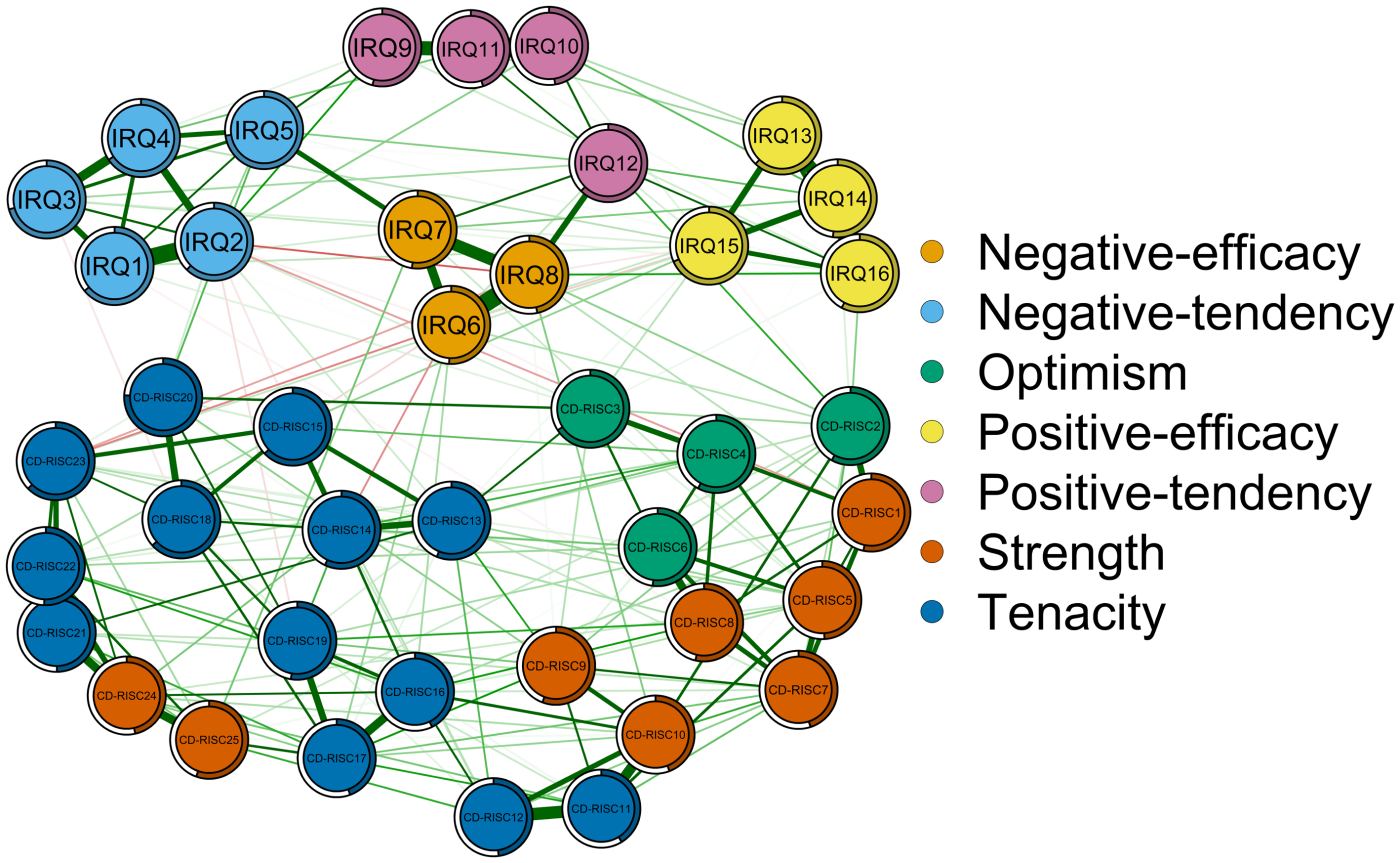
Network structure of interpersonal emotional regulation and psychological resilience of nursing undergraduates. Note: Green edges represent positive associations and red edges represent negative associations between nodes. The thickness of an edge is proportional to the strength of the association.

Figure 1 shows the network architecture of interpersonal emotional regulation and psychological resilience. The strongest association in the interpersonal emotion regulation network is IRQ6 (Appreciate support)—IRQ8 (Appreciate help). The strongest associations in the psychological resilience network is CD-RISC11 (Believe in achieving goals)—CD-RISC12 (do not give up). The strongest edges of the interpersonal emotional regulation—psychological resilience network are IRQ12 (Celebrate the good things)—CD-RISC2 (intimate relationship), IRQ5 (need attention)—CD-RISC20 (Follow one’s instincts) and IRQ7 (Need to care)—CD-RISC25 (Take pride in achievement) (Supplementary Table S1). CD-RISC20 (Follow one’s instincts) was the most predictable (0.758), while CD-RISC11 (Believe in achieving goals) was the least predictable (0.413).

**Figure 2:**
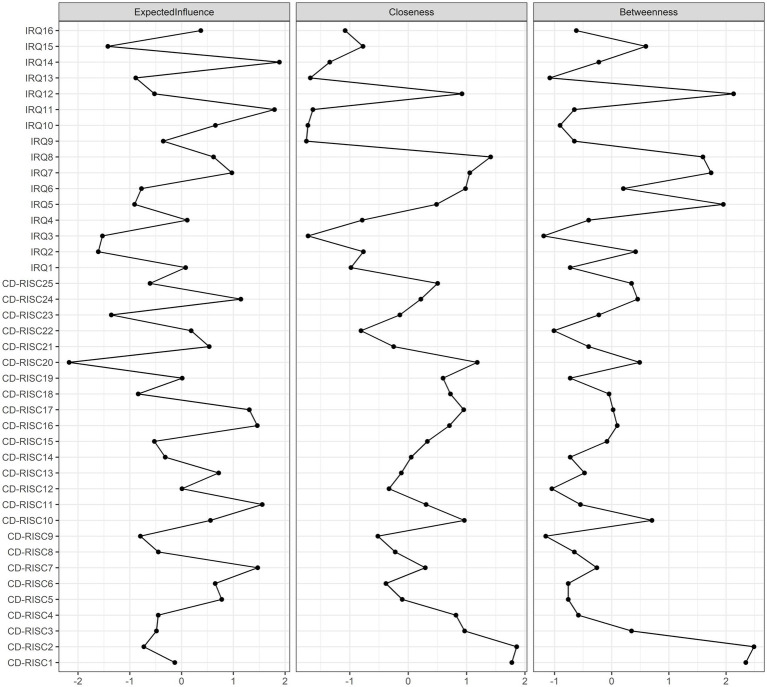
Centrality plot depicted the expected influence (z-score) of each variable chosen in the final network.

The Expected Impact Index (Figure 2) indicates that IRQ14 (Enjoy being with friends), IRQ11 (Like to share), and CD-RISC11 (Believe in achieving goals) are core nodes, with IRQ14 having the highest centrality.

**Figure 3:**
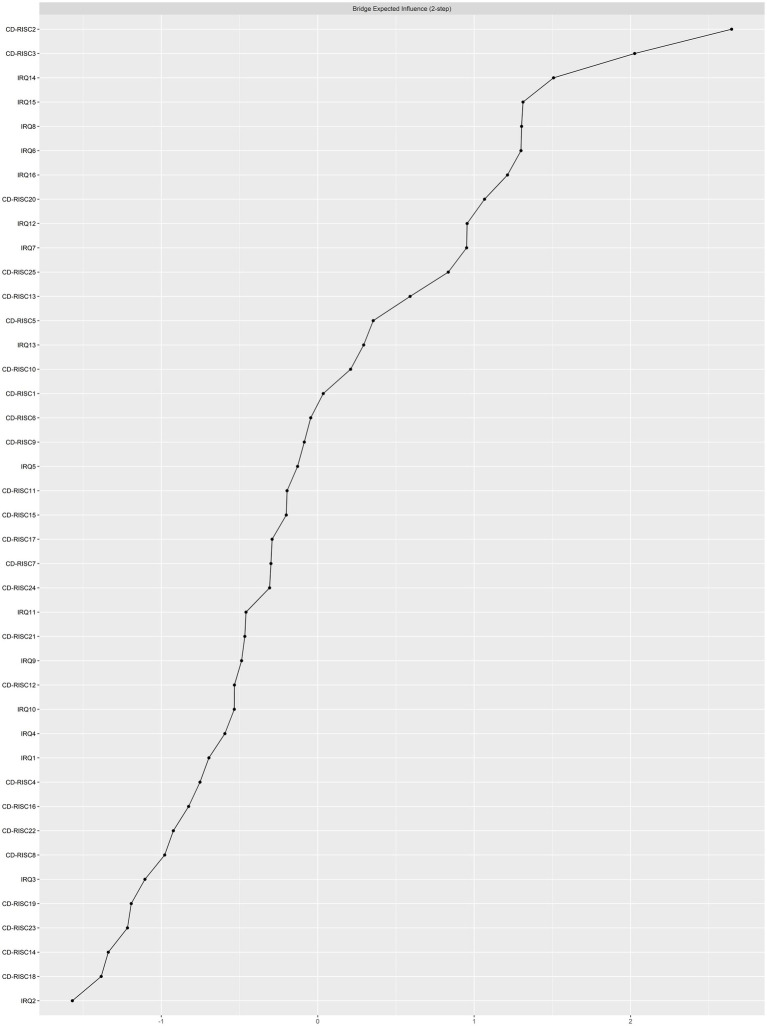
Centrality plot depicted the bridge expected influence (z-score) of each variable chosen in the final network.

The bridge expected impact index (Figure 3) shows IRQ14 (Enjoy being with friends), CD-RISC2 (intimate relationship), and CD-RISC3 (The help of fate) are the bridging nodes between interpersonal emotion regulation node cluster and psychological resilience node cluster. “Enjoy being with friends” is from IRQ16, and “intimate relationship” and “The help of fate” are from CD-RISC25. This suggests that “Enjoy being with friends” may affect. The level of psychological resilience of college students, while “intimate relationship” and “The help of fate” affect the interpersonal emotional regulation ability of college students.

The interpersonal emotional regulation-psychological resilience network showed good stability. The 95% confidence interval for bootstrap indicates that the accuracy of the edge weights is relatively reliable and accurate (Supplementary Figure S1). In addition, the CS coefficients of the expected impact and bridge expected impact are 0.75 and 0.516, respectively, indicating that the estimates of the expected impact and bridge expected impact are sufficiently stable (Supplementary Figures S2). The bootstrapped difference test for edge weights were shown in Supplementary Figure S3. The bootstrapped difference test for node expected influences were shown in Supplementary Figure S4.

In the network structure of senior medical students, the strongest edge is IRQ8 (Appreciate help) -CD-RISC2 (intimate relationship). IRQ14 (Enjoy being with friends), IRQ11 (Like to share) and CD-RISC11 (Believe in achieving goals) are core nodes. IRQ15 (Stay around other people), CD-RISC2 (intimate relationship), and CD-RISC3 (The help of fate) are the highest bridge nodes (Supplementary Figure S5). In the network structure of junior medical students, the strongest edge is IRQ12 (Celebrate the good things)—CD-RISC2 (intimate relationship). IRQ14 (Enjoy being with friends), CD-RISC16 (Not easily defeated), and IRQ11 (Like to share) are core nodes. IRQ14 (Enjoy being with friends), CD-RISC2 (intimate relationship), and CD-RISC3 (The help of fate) are the highest bridge nodes (Supplementary Figure S6).

## 4 Discussion

In this study, we used network analysis to explore the network structure of interpersonal emotional regulation and psychological resilience in a group of nursing undergraduates. In this network, we find a strong relationship between IRQ6 (Appreciate support) and IRQ8 (Appreciate help). The reason may be that both of them belong to the negative emotion efficacy dimension of interpersonal emotion regulation. We found a strong association between CD-RISC11 (Believe in achieving goals) and CD-RISC12 (do not give up), both belong to the tenacity dimension of psychological resilience. We also found a strong association between IRQ12 (Celebrate the good things) and CD-RISC2 (intimate relationship). “Celebrate the good things” shows that when I want to celebrate some good things, I will go to certain people to share them. It belongs to the positive emotion tendency dimension in interpersonal emotion regulation, which refers to people’s tendency to increase their positive emotions. The expression of “intimate relationship” is to have some close and stable relationships, including relatives, friends, lovers, etc., which reflects that people have good interpersonal relationships. Our study found an association between the two, which may reflect that they share the same underlying influencing factors, which is consistent with previous findings (Wei et al., 2023; Zhang et al., 2023). Previous studies have shown that interpersonal adaptation can significantly positively predict positive emotions, and interpersonal sensitivity can affect the generation of positive emotions. Interpersonal sensitivity refers to the tendency to be alert and sensitive to others’ evaluation and to take defensive actions to avoid negative evaluation, which itself has a certain negative tendency. When people show interpersonal sensitivity, it will prevent individuals from receiving love, understanding and support from the outside world, thus affecting the production of positive emotions (Zhang et al., 2023). In addition, positive emotions also promote good interpersonal interaction. Positive emotions can provide individuals with supportive psychological resources and increase their sense of interpersonal control. The extension-construction theory holds that positive emotions have evolutionary adaptive value to individuals, and positive emotions can immediately expand the scope of individual thought-action, and on this basis, construct more lasting psychological, physiological and social resources to help individuals obtain long-term satisfactory relationships in social interactions (Wei et al., 2023; Gao and Tong, 2010). This also makes the two constitute a mutually reinforcing feedback loop.

We found that IRQ14 (Enjoy being with friends), IRQ11 (Like to share), and CD-RISC11 (Believe in achieving goals) were core nodes in the interpersonal emotional regulation-psychological resilience network. This suggests that these symptoms may play an important role in activating and maintaining the interpersonal emotional regulation-psychological resilience network. In the intervention process, researchers will use these three nodes as a target for the development of intervention strategies, may get greater benefits. “Enjoy being with friends” means smiling when you are with others. “Like to share” means that when things are going well, one feels compelled to share with others. Both nodes are reflected in a person’s ability to have good relationships and increase positive emotions. Previous studies have shown that students who are more popular among people have a higher ability of emotional regulation (Gross and John, 2003; Zhou et al., 2021), and interpersonal relationships also have a significant positive impact on psychological resilience (Zheng et al., 2023). Students with good interpersonal relationships can strengthen their environmental protection factors and thus obtain a higher level of psychological resilience. Positive emotions have also been shown to positively predict people’s interpersonal emotional regulation behaviors and levels of psychological resilience (Xiong and Li, 2024; Lyzwinski et al., 2024). Therefore, in the intervention, we can achieve precise positioning and intensive intervention on interpersonal relationships and the ability to express positive emotions, so as to improve students’ interpersonal emotional regulation ability and psychological resilience level. CD-RISC11 (Believe in achieving goals) is another core node in the Interpersonal Emotional regulation-Psychological resilience network. “Believe in achieving goals” means that I believe I can achieve my goals despite obstacles, which reflects the degree of confidence of an individual in the behavioral ability to complete a specific task, that is, the individual self-efficacy. Our study identifies the central role of self-efficacy in the interpersonal emotion regulation-psychological resilience network, which is consistent with previous findings. Previous studies have shown that self-efficacy can effectively predict people’s emotional regulation ability and psychological resilience level (Xu et al., 2023; Wu and Man, 2018). Individuals with higher self-efficacy tend to believe that they can cope with various challenges, tend to maintain a resilient attitude in the face of difficulties, and thus the easier it is to adopt positive emotion regulation strategies to solve problems. In recent years, emotional regulation self-efficacy, as a part of general self-efficacy, has become a new hot topic of research, which refers to the degree of confidence that an individual can manage their emotions (Schunk et al., 2022). Many researchers have begun to pay attention to the relationship between emotional regulation self-efficacy, negative emotions and psychological resilience (Wu and Man, 2018). Therefore, it is of great significance to improve students’ self-efficacy for their mental health.

The results of core nodes suggest that nursing educators should focus on students’ interpersonal relationships, emotional states and self-efficacy. First of all, schools should strengthen the construction of mental health education teachers, reasonably equip professional psychological counselors according to the number of students, and provide personalized and targeted psychological counseling services for students in interpersonal relationships, emotional states, self-efficacy and other aspects. For example, the implementation of solution-focused group counseling (SFGC) can effectively improve students’ interpersonal relationships (Franklin and Hai, 2021). Through focusing on the main technical questions, group empowerment and providing a positive environment, we can improve students’ conversation skills and relationship handling ability, promote students’ proactive communication, and help students establish stable interpersonal relationships. Dialectical behavior therapy group counseling, narrative therapy, dancing therapy are also effective ways to improve students’ interpersonal relations (Guo et al., 2024; Xu, 2022; Guo, 2020). Positive psychological intervention may be a good choice to cultivate students’ positive emotions (Lyzwinski et al., 2024). Positive Psychological Interventions (PPI) are guided by the ideas of positive psychology and are designed to help individuals discover, strengthen, and sustain the “positive resources” in their lives to develop positive emotions, behaviors, or values. Mindfulness group training, self-affirmation training, spiritual care and suggestion therapy have a good effect on improving students’ self-efficacy (Zhou et al., 2024; Zhu and Li, 2022). Secondly, in addition to professional psychological intervention, schools should strengthen the ability of counselors and teachers to provide mental health services, integrate mental health education into professional teaching, and create an optimistic and positive learning and career atmosphere for students. In addition, nursing educators can use the Internet and information platform to change the traditional teaching mode of “knowledge infusion” and actively carry out social practice of nursing professional mental health education, including competitions, forums, and situational simulation activities. Through flexible curriculum practice activities, help students to establish a good psychological cognition, establish positive values, so as to fundamentally enhance the professional interest of nursing students, cultivate students’ professional identity and pride.

Our study identified 3 of the strongest bridging nodes between interpersonal emotional regulation and psychological resilience. These include IRQ14 (Enjoy being with friends), CD-RISC2 (intimate relationship), and CD-RISC3 (The help of fate). Bridge node means that the node has a connection effect between different node groups in the network structure, and it has an important influence on other node groups in the network. In interpersonal emotional regulation, “Enjoy being with friends” is the strongest bridge node, which indicates that the intervention aimed at interpersonal relationships may have greater benefits on the level of psychological resilience of college students, which validates the previous findings to a certain extent. A survey of 471 college students shows that the emotional regulation ability and psychological resilience of college students are regulated by interpersonal relationships. Interpersonal relationships regulate self-efficacy by expressing positive emotions and manage negative self-efficacy play an incomplete mediating role in psychological resilience (Wu and Man, 2018). Therefore, focusing on students’ interpersonal relationship (including dormitory relationship, peer relationship and teacher-student relationship) has a certain activation effect on psychological resilience. The other two bridge nodes are “intimate relationship” and “The help of fate” in psychological resilience. “intimate relationship” means having some close and stable relationships (relatives, friends, lovers, etc.); “intimate relationship” means that when my problems cannot be clearly solved, sometimes fate or chance will help me; These two nodes belong to the optimistic dimension of psychological resilience, reflecting the degree of optimism of the individual. This indicates that individual optimism also has a certain connection effect on interpersonal emotion regulation and psychological resilience, and will have a certain impact on people’s interpersonal emotion regulation ability. Optimism refers to an individual’s greater belief that events will turn out for the better. Some studies have found that optimists have the flexibility to choose a variety of emotional regulation strategies in the face of negative emotions. In addition, personality traits have been proved to be predictors of individual interpersonal emotion regulation ability, and optimism, as a positive personality trait, plays an important role in people’s interpersonal emotion regulation ability (Wang and Wang, 2023). Therefore, nursing educators can improve students’ psychological resilience from the perspective of improving interpersonal relationship. Starting from the aspects of establishing positive life cognition, enhancing positive emotional experience and cultivating positive behavior tendency, this paper cultivates students’ optimistic personality traits through rational attribution and positive psychological intervention, so as to improve students’ interpersonal emotion regulation ability.

In our study, we found some differences in the structure of the interpersonal emotional regulation-psychological resilience network between upper and lower grades. In the upper-level network architecture, the strongest edge is IRQ8 (Appreciate help) with CD-RISC2 (intimate relationship), while the whole crowd and lower grades are IRQ12 (Celebrate the good things) with CD-RISC2 (intimate relationship). “Appreciate help” belongs to the negative emotion efficacy dimension in interpersonal emotion regulation, and “Celebrate the good things” belongs to the positive emotion tendency dimension in interpersonal emotion regulation, indicating that senior students tend to maintain a stable relationship by reducing negative emotions. Lower grade students were more likely to maintain stable relationships by increasing positive emotions. However, Ni Ying’s study found that juniors were more likely to use positive emotion regulation strategies (cognitive reappraisal) than sophomores. The reason for the two different results may be the different emphasis of psychological education in different schools. In Ni Ying’s study, senior students may have more positive changes in emotion regulation after school training (Ni, 2024). Also, in the network structure of the lower grades, unlike the whole population and the upper grades, CD-RISC16 (Not easily defeated) was identified as the core node, rather than CD-RISC11 (Believe in achieving goals). This may be because lower grade students are still in the stage of adapting to the new school environment, and stress resistance plays a greater role in psychological resilience, while senior students are currently in the stage of self-realization, and self-efficacy has greater significance in psychological resilience. This is consistent with the findings of Kotter et al., which show that stress tolerance among first-year medical students is an important predictor of their own mental health (Kötter et al., 2016). Therefore, for students of different grades, nursing educators should start from different angles to provide personalized mental health services for students, so as to achieve better results.

## 5 Limitations

There are still some limitations to our study. First of all, our study is a cross-sectional study, which is difficult to reveal the dynamic change relationship between different components, and cannot draw causal conclusions. Future studies should use longitudinal or experimental design to examine the temporal causal relationship. Second, in the network analysis, we only studied the variables we chose, and since the results are highly variable dependent, the results may change somewhat if other variables are included in the network. In addition, variable measurements were self-reported, and the results may be biased, so all results should be interpreted with caution, and other relevant variables can be added in the future to test the more specific relationship between emotional regulation and psychological resilience. Third, the study population was nursing students from China, and females accounted for a large proportion, which may not be applicable to other populations. Future studies should verify whether these findings can be extended to other populations.

## 6 Conclusion

In conclusion, our study outlines the multi-level and multi-dimensional interactions among the components of the interpersonal emotional regulation-psychological resilience network structure of nursing undergraduates. “Enjoy being with friends,” “enjoy sharing” and “have confidence to achieve goals” were identified as the key core nodes, which indicates that nursing educators from the perspective of students’ interpersonal relationship, emotional state and self-efficacy, to provide students with targeted psychological intervention, may have a better effect on improving students’ interpersonal emotional regulation ability and psychological resilience level. “Enjoy being with friends,” “intimate and stable relationship” and “help of fate” were identified as the key bridge nodes, which indicates that nursing educators can give full play to the connecting role of bridge nodes to improve students’ psychological resilience from the perspective of improving interpersonal relationships. From the perspective of cultivating students’ optimistic quality, improve students’ interpersonal emotion regulation ability. We should also note that there may be some differences in the core node focus of students’ interpersonal emotional regulation skills and psychological resilience levels at different grades, and that individualized mental health services by nursing educators may achieve better results for students at different grades. The correlation revealed in this study may provide some help for the precise intervention of interpersonal emotional regulation ability and psychological resilience of nursing undergraduates, but more confirmatory studies are still needed to verify it.

## Data Availability

The original contributions presented in the study are included in the article/Supplementary material, further inquiries can be directed to the corresponding author.
