# Supplementary material for: The relationship between interpersonal emotional regulation and psychological resilience in nursing undergraduates: a cross-sectional study and network analysis
Source: Front Psychol. 2025 Mar 7;16:1546042. doi: 10.3389/fpsyg.2025.1546042 (PMC11925865; doi:10.3389/fpsyg.2025.1546042)
Supplement: Supplementary file 1 [file Supplementary_file_1.doc]

**Supplementary Materials**

1. TableS1 Nonparametric Spearman rho correlation matrix of interpersonal emotional regulation and psychological resilience.
2. Figure S1 Accuracy of edge weights.
3. FigureS2 Stability of node expected influences and node bridge expected influences.
4. Figure S3 Bootstrapped difference test for edge weights.
5. Figure S4 Bootstrapped difference test for node expected influences.
6. Figure S5 Network structure of interpersonal emotional regulation and psychological resilience of Senior medical student.
7. Figure S6 Network structure of interpersonal emotional regulation and psychological resilience of junior medical student.

TableS1 Nonparametric Spearman rho correlation matrix of interpersonal emotional regulation and psychological resilience


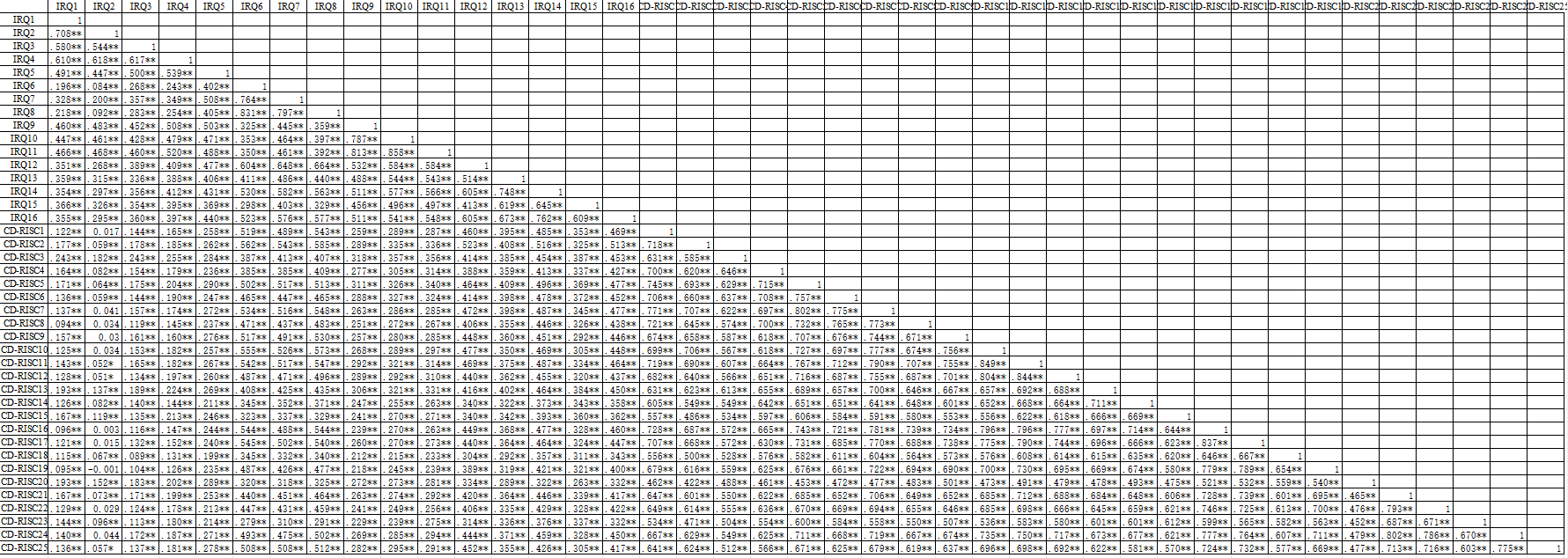





Figure S1 Accuracy of edge weights.

Note: The red line represents the edge, as estimated in the sample. The grey indicates 95% bootstrapped confidence interval.





Figure S2 Stability of node expected influences and node bridge expected influences.

*Note*: The **purple** bar represents the average correlation between expected influence in the full sample and subsample with the **purple** area depicting the 2.5th quantile to the 97.5th quantile. The green bar represents the average correlation between bridge expected influence in the full sample and subsample with the green area depicting the 2.5th quantile to the 97.5th quantile.





Figure S3 Bootstrapped difference test for edge weights.

Note: Gray boxes indicate edge weights that do not differ significantly from one another, while black boxes indicate edge weights that do differ significantly. Blue and red boxes on the diagonal correspond to edge weights with positive and negative correlations, respectively.





Figure S4 Bootstrapped difference test for node expected influences.

*Note*: Gray boxes indicate node expected influences that do not differ significantly from one another, while black boxes indicate node strengths that do differ significantly. The number in the white boxes (i.e., diagonal line) represent the value of node strengths.


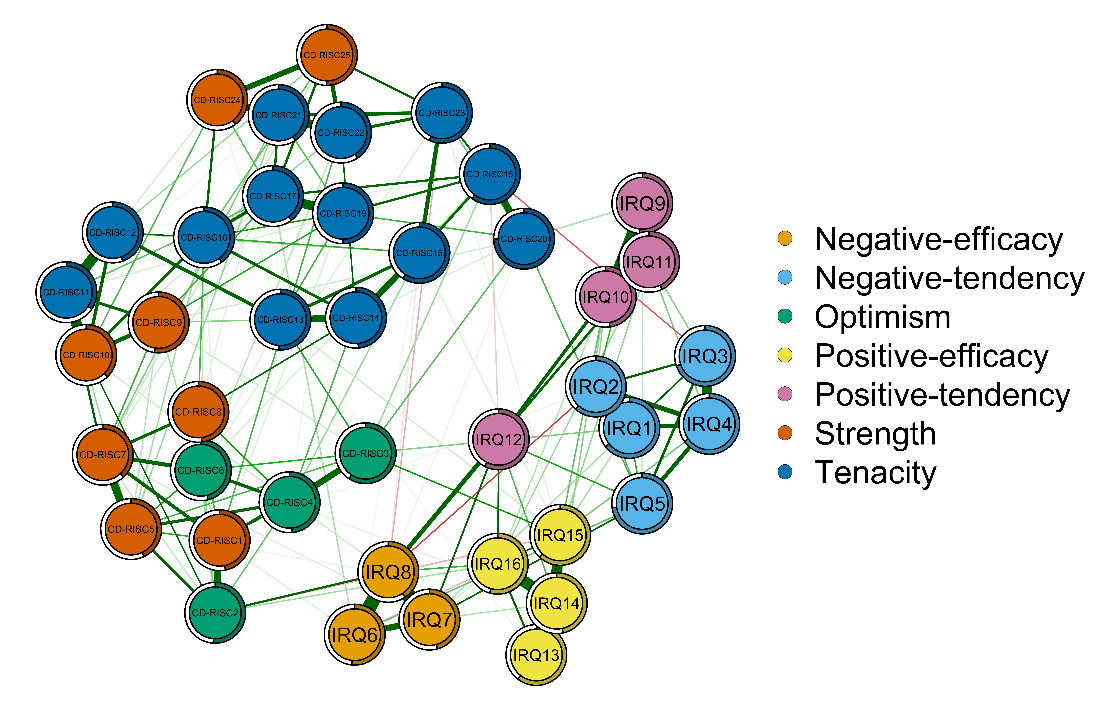


Figure S5 Network structure of interpersonal emotional regulation and psychological resilience of Senior medical student.

Note: Green edges represent positive associations and red edges represent negative associations between nodes.


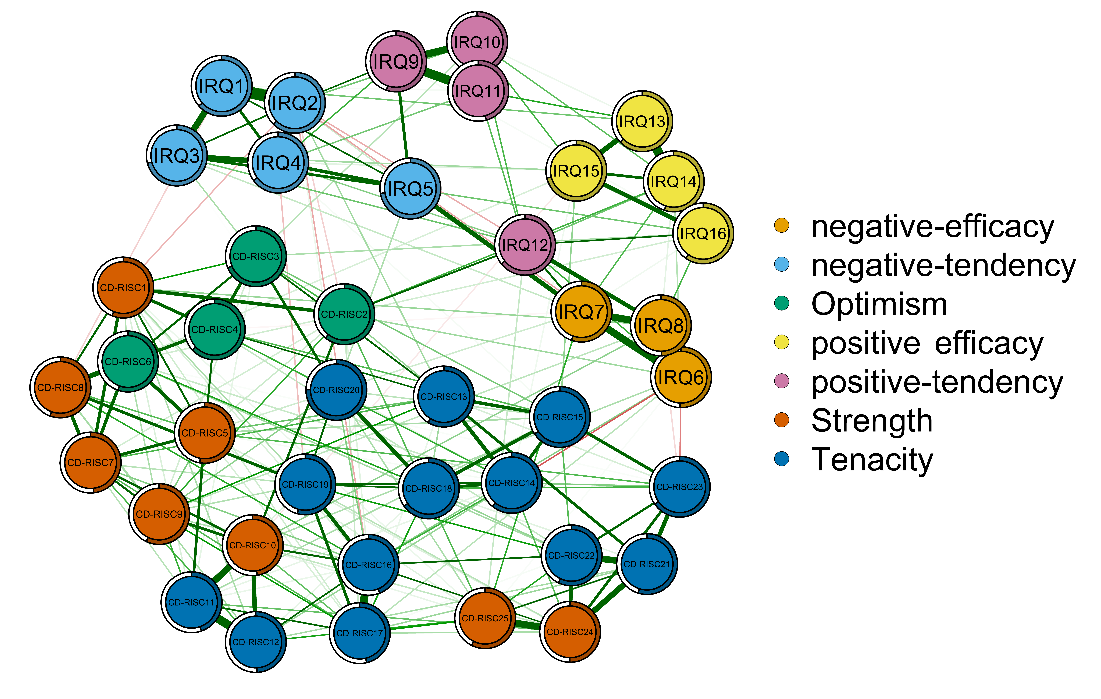


Figure S6 Network structure of interpersonal emotional regulation and psychological resilience of junior medical student.

Note: Green edges represent positive associations and red edges represent negative associations between nodes.
